# The complete mitochondrial genome of yellow crazy ant, *Anoplolepis gracilipes* (Hymenoptera: Formicidae)

**DOI:** 10.1080/23802359.2018.1467739

**Published:** 2018-05-17

**Authors:** Chih-Chi Lee, John Wang, Kenji Matsuura, Chin-Cheng Scotty Yang

**Affiliations:** a Laboratory of Insect Ecology, Graduate School of Agriculture, Kyoto University, Kyoto, Japan;; b Research Institute for Sustainable Humanosphere, Kyoto University, Kyoto, Japan;; c Biodiversity Research Center, Academia Sinica, Taipei, Taiwan

**Keywords:** Yellow crazy ant, mitochondrial genome, invasive ant

## Abstract

The yellow crazy ant *Anoplolepis gracilipes* is an invasive species that threatens biodiversity in introduced ecosystems. We sequenced the *A. gracilipes* mitogenome using next-generation sequencing methods. The circular mitogenome of *A. gracilipes* was 16,943 bp included 13 protein-coding genes, two ribosomal RNA genes, 22 transfer RNAs, and a single large non-coding region of 893 bp. The base composition was AT-biased (72%). Three genomic rearrangements compared to ancestral insects were found. Phylogenetic analysis based on the concatenated nucleotide sequences of the 13 protein-coding genes supports *A. gracilipes* belonging to the Formicinae subfamily. We announce the *A. gracilipes* mitogenome as a DNA reference for further population genetic, phylogenetic, and evolutionary analyses.

The yellow crazy ant (*Anoplolepis gracilipes*) is a widespread invasive species of great conservation concern (O’Dowd et al. 2003). This ant is polygynous (multiple-queens) and often forms supercolonies (Hoffmann and Hagedorn 2014). Colonies usually reproduce by dependent foundation although independent foundation also was observed (Ito et al. 2016). The mitochondrial DNA has been used as a genetic marker to characterize the population structure of this ant (Thomas et al. 2010; Gruber et al. 2012), however, no complete mitochondrial genome is currently available. Here, we present the first complete mitogenome for this species.

Specimens of *A. gracilipes* were collected from Hsinchu City, Taiwan (24**°**46′43.43″N, 120**°**56′30.24″E) and preserved in 70% EtOH (archived in the Laboratory of Ecosystem Management and Conservation Ecology, No. Ano062), Kyoto University, Kyoto, Japan. We extracted genomic DNA from a single virgin queen via the Phenol–Chloroform purification method. We sequenced DNA on the Illumina HiSeq^TM^ 2500 platform with PCR-free library construction (average library insert size: 250 bp; paired-end read length: 126 bp).

For assembly/annotation, we removed adapters from raw sequence reads with Trimmomatic v0.36 (Bolger et al. 2014) and then conducted *de novo* mitogenome assembly with NOVOPlasty v2.6.4 (using the *Camponotus atrox* mitogenome as the seed) (Dierckxsens et al. 2016). Average read coverage of the mitogenome assembly was 4,849, providing ample depth for correctness. We annotated protein coding genes (PCGs), rRNAs, and tRNAs using MITOS (Bernt et al. 2013), OrfFinder (Coordinators 2016), and ARWEN (Laslett and Canbäck 2007).

Similar to other ant mitogenomes, the complete mitogenome of *A. gracilipes* (GenBank: MH122734) is 16,943 bp. The nucleotide composition is AT-biased (72%). The mitogenome contains 13 PCGs, two rRNAs, and 22 tRNAs, typical for most animals. The tRNAs, ranging in size from 59 to 75 bp, are similar to other ants (circa 54–90 bp). The gene order (GO) has three rearrangements compared to the ancestral insect (ancestor GO: *A. gracilipes* GO; trnR trnN trnS1 trnE: trnR trnS1 trnN trnE; -*nad4* -*nad4L* trnT -trnP: -*nad4* trnT -*nad4L* -trnP; CR trnI -trnQ trnM *nad2*: CR trnM trnI -trnQ *nad2*). The first two GO rearrangements differed from two *Formica* species (Babbucci et al. 2014; Yang et al. 2016), whereas all the three rearrangements differed from *Camponotus atrox* and *Polyrhachis dives* (Kim et al. 2015; Liu et al. 2017). Such differences in GO rearrangement are consistent with the tribe-level classification of these species where *Anoplolepis*, *Formica* and *Camponotus*/*Polyrhachis* belong to Plagiolepidini, Formicini and Camponitini, respectively (Blaimer et al. 2015). All PCGs use ATN as the start codon (N, any nucleotide) and TAA as the stop codon. The control region presumably corresponds to the single largest non-coding AT-rich region (893 bp, A+T 93%).

We inferred the phylogenetic relationship of 16 ants and two bees, using the concatenated nucleotide sequences of all 13 PCGs. Both Bayesian inference (MrBayes 3.2.5, GTR+I+Γ model (Ronquist et al. 2012)) and maximum likelihood (RAxML v8.2.11, GTRGAMMA model (Stamatakis 2014)) computation agree with the current phylogenetic placement of *Anoplolepis* in the Formicinae (Figure 1).

**Figure 1.**
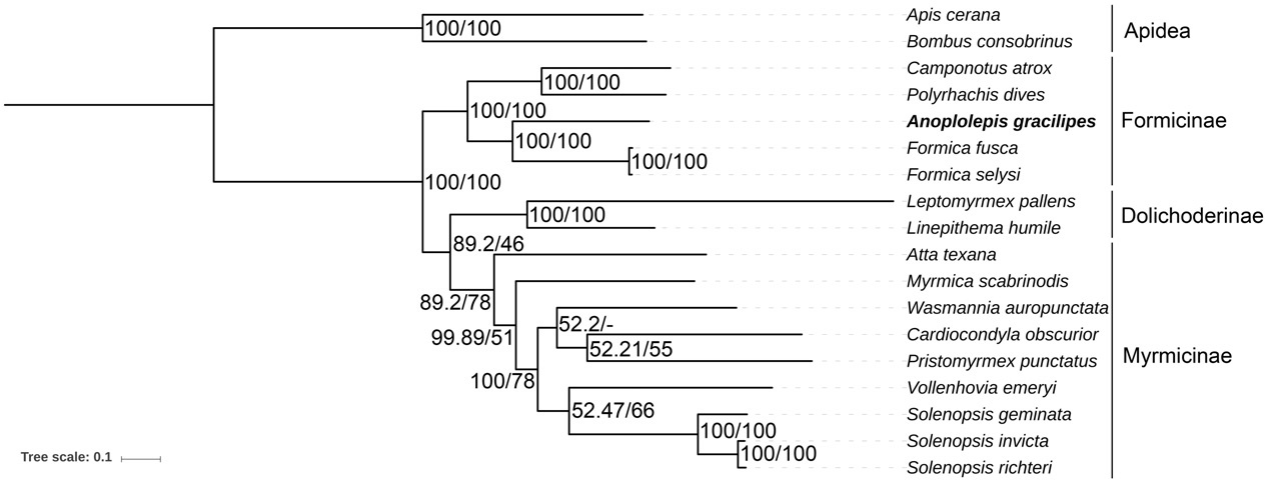
Molecular phylogeny of *Anoplolepis gracilipes* and 17 other Hymenoptera species (15 ants and two bees) based on the concatenated nucleotide sequences of 13 PCGs. The phylogenetic tree was constructed by the Bayesian inference and Maximum likelihood methods under GTR + I + Γ and GTRGAMMA models, respectively. The numbers at each node indicate the posterior probability (100,000 generations, sampled every 100 generations) and the bootstrap probability (1,000 replicates) resulting from the analyses. Note that only Bayesian probability is shown at the branch node of *Wasmannia auropunctata* as slight differences in placement of this species were observed between the two phylogenetic methods. The mitogenome accession numbers for the tree construction are listed as follows: *Apis cerana* (GQ162109), *Atta texana* (MF417380), *Bombus consobrinus* (MF995069), *Camponotus atrox* (KT159775), *Cardiocondyla obscurior* (KX951753), *Formica fusca* (LN607805), *F. selysi* (KP670862), *Leptomyrmex pallens* (KC160533), *Linepithema humile* (KX146468), *Myrmica scabrinodis* (LN607806), *Polyrhachis dives* (NC_030790), *Pristomyrmex punctatus* (NC_015075), *Solenopsis geminata* (NC_014669), *S. invicta* (NC_014672), *S. richteri* (HQ215539), *Vollenhovia emeryi* (NC_030176), *W. auropunctata* (KX146469). *We rooted the phylogenetic tree at *A. cerana* and *B. consobrinus* (two bees) based on their exclusion from Formicidae (ants).
